# Comprehensive evaluation of soil quality in a desert steppe influenced by industrial activities in northern China

**DOI:** 10.1038/s41598-021-96948-7

**Published:** 2021-09-01

**Authors:** Zhe Xu, Wenbao Mi, Nan Mi, Xingang Fan, Yao Zhou, Ying Tian

**Affiliations:** 1 College of Agriculture, Ningxia University, Yinchuan, 750021 Ningxia China; 2 School of Geography and Planning, Ningxia University, Yinchuan, 750021 China; 3 West Development Research Center, Ningxia University, Yinchuan, 750021 China

**Keywords:** Agroecology, Grassland ecology, Environmental impact

## Abstract

Desert steppe soil security issues have been the focus of attention. Therefore, to understand the impact of industrial activities on the soil quality of desert grasslands, this experiment investigated the Gaoshawo Industrial Concentration Zone in Yanchi County. Based on the distance and direction from the industrial park, sample plots were established at intervals of 1–2 km. A total of 82 surface soil samples (0–20 cm) representing different pollution sources were collected. The samples were analysed for pH, total nitrogen, total phosphorus, available phosphorus, available potassium, organic matter, copper (Cu), cadmium (Cd), chromium (Cr), lead (Pb), and zinc (Zn). The desert steppe soil quality was analysed based on the integrated fertility index (IFI) and the Nemerow pollution index (PN), followed by the calculation of the comprehensive soil quality index (SQI), which considers the most suitable soil quality indicators through a geostatistical model. The results showed that the IFI was 0.393, indicating that the soil fertility was relatively poor. Excluding the available potassium, the nugget coefficients of the fertility indicators were less than 25% and showed strong spatial autocorrelation. The average values of Cu, Cd, Cr, Pb and Zn were 21.64 ± 3.26, 0.18 ± 0.02, 44.99 ± 21.23, 87.18 ± 25.84, and 86.63 ± 24.98 mg·kg^−1^, respectively; the nugget coefficients of Cr, Pb and Zn were 30.79–47.35%. Pb was the main element causing heavy metal pollution in the study area. Higher PN values were concentrated north of the highway in the study area, resulting in lower soil quality in the northern region and a trend of decreasing soil quality from south to north. The results of this research showed that the average SQI was 0.351 and the soil quality was extremely low. Thus, industrial activities and transportation activities in the Gaoshawo Industrial Zone significantly impact the desert steppe soil quality index.

## Introduction

With the continuous increase in the average global temperature, soil quality has been a focus of research regarding climatic warming. Spatial differences in soil quality are affected not only by natural factors but also by land use, human activities, and agricultural management^1^. In recent decades, urbanization and industrialization in China have developed at unprecedented rates^2^. As the centre of Chinese coal mining has shifted from central China to the northwest, large-scale, high-intensity coal mining activities have further threatened the fragile eco-environment of northwestern China^3^. Soil quality can be defined as “the ability of soil to sustain plant and animal productivity, maintain or enhance water and air quality, and support human health and habitats in natural and managed ecosystems”^4,5^. Heavy metals and other pollutants are easily released in the processes related to industrial production, transportation, combustion, etc. These pollutants do not degrade easily, have poor fluidity, and accumulate easily. When excessive heavy metals enter the soil, the quality of the soil decreases due to a decrease in soil productivity^6,7^. Industrial production undermines the land in arid steppes with scarce topsoil, especially in mining areas^8^. Therefore, the focus on and accurate evaluation of soil quality is important to prevent soil degradation.

Due to the complexity of factors affecting soil quality, there is no unified evaluation system for assessing soil quality^9^. At present, most studies measure soil quality status by evaluating the physical, chemical, and biological characteristics of the soil^10^. The main evaluation methods include multiple linear regression analysis^11^, principal component analysis^12,13^, minimum data set (MDS) and revised minimum data set (RMDS). Most studies on soil quality indexes (SQIs) have focused on wetlands, cultivated land, and farmland^14–17^, but few have focused on grassland soil quality^18^. SQIs are important for evaluating grassland ecosystems. Zhou et al.^19^ analysed the soil qualities of different land uses using a total data set, MDS and RMDS indicator selection methods and linear and non-linear scoring methods. Cheng et al.^20^ analysed the content and source of heavy metals in the grassland near the Shengli coal mine base and found that the average concentration of heavy metals was low and did not threaten the local soil quality, although the coal base had a significant effect on the heavy metal concentration in the grassland. On the Qinghai-Tibet Plateau, Zhao et al.^21^ analysed the physical and chemical properties of the soil, used a comprehensive fuzzy mathematical model combined with the soil quality index to evaluate and calculate the soil quality in the study area, and found that the grassland soil quality was low. The evaluation indexes and methods selected differed among the study areas due to the influence of the evaluation scale, soil, climate and other factors. Therefore, two important factors, soil fertility and soil environment, should be considered when analysing soil quality^22^.

Ningxia is located in the eastern part of northwestern China and has an arid climate. The grassland types in this area are mainly desert grasslands and steppe, which are distributed in southern and eastern Ningxia. Coal, oil, and natural gas resources are located in the area. Industrial activities centred on the development and processing of these natural resources have greatly promoted local economic development but have negatively impacted the environment. The fragile ecological environment and low carrying capacity of the ecosystems in the region make it susceptible to grassland degradation by improper resource development and utilization. For a long time, grassland soil has been affected by fossil fuel combustion, industrial emissions, construction dust, vehicle exhaust emissions, and air particulates, which are likely to cause the accumulation of heavy metals in the soil^23,24^. Therefore, heavy metal pollution has become an important factor to consider when evaluating desert steppe soil quality.

In this research, we use geostatistics and fuzzy mathematics to analyse the physical and chemical properties of soil and the content of heavy metals in soil to further understand the impact of industrial activities on the quality of desert grassland soil. It is assumed that the content of heavy metals in the soil has a negative impact on soil fertility and soil quality. To test this hypothesis, the purpose of this study is to (1) determine the soil fertility index (IFI) through principal component analysis, (2) use the Nemerow pollution index to determine the soil pollution index (PN) of the research area, and (3) construct a comprehensive soil fertility index and internal Nemerow pollution index to comprehensively evaluate soil quality, hoping to provide support for the protection and healthy development of desert grasslands in the future.

## Materials and methods

### Overview of the study area

The study area is located west of Gaoshawo town, Yanchi County, Ningxia (106° 49′ 6.18′′ E, 38° 07′ 9.93′′ N) and is connected with the Mu Us Desert in the Etuokeqian Banner of the Inner Mongolia Autonomous Region in northern China. It covers an area of 80.46 km^2^ at an altitude of 1409 m and has a moderate temperate continental climate, with cold winters, hot summers, an average temperature of 22.4 °C, and annual average precipitation of 276 mm. The region is mainly composed of gentle slopes and hills and contains a large amount of oil, coal, natural gas, and other resources. The soil types are mainly calcareous and aeolian sandy soils, with loose particle structure and low concentrations of organic matter and nutrients. According to the second soil census in China and the nutrient grading standard^25^, the levels of the soil nutrients in the study area were relatively poor, low, and moderate. The soil nitrogen fixation ability is low, and there is severe phosphorus loss. The region is dominated by steppe, desert steppe, and sandy vegetation. The major vegetation types in the area include *Stipa breviflora* Griseb., *Agropyron cristatum* (L.) Gaertn., *Pennisetum centrasiaticum* Tzvel., *Lespedeza potaninii* Vass., *Potentilla chinensis* Ser., and *Artemisia scoparia* Waldst. et Kit. The study area, which is the main area for industrial production and raw material transportation, is crossed by the Qingyin Expressway, 307 National Road, and the Taiyin Railway. The main types of industries in the park are coal chemicals, gasoline chemicals and gypsum deep processing. According to a survey, 18–20 vehicles involved in coal transportation travel in and out of the industrial zone daily.

### Sample collection and characterization

**Figure 1:**
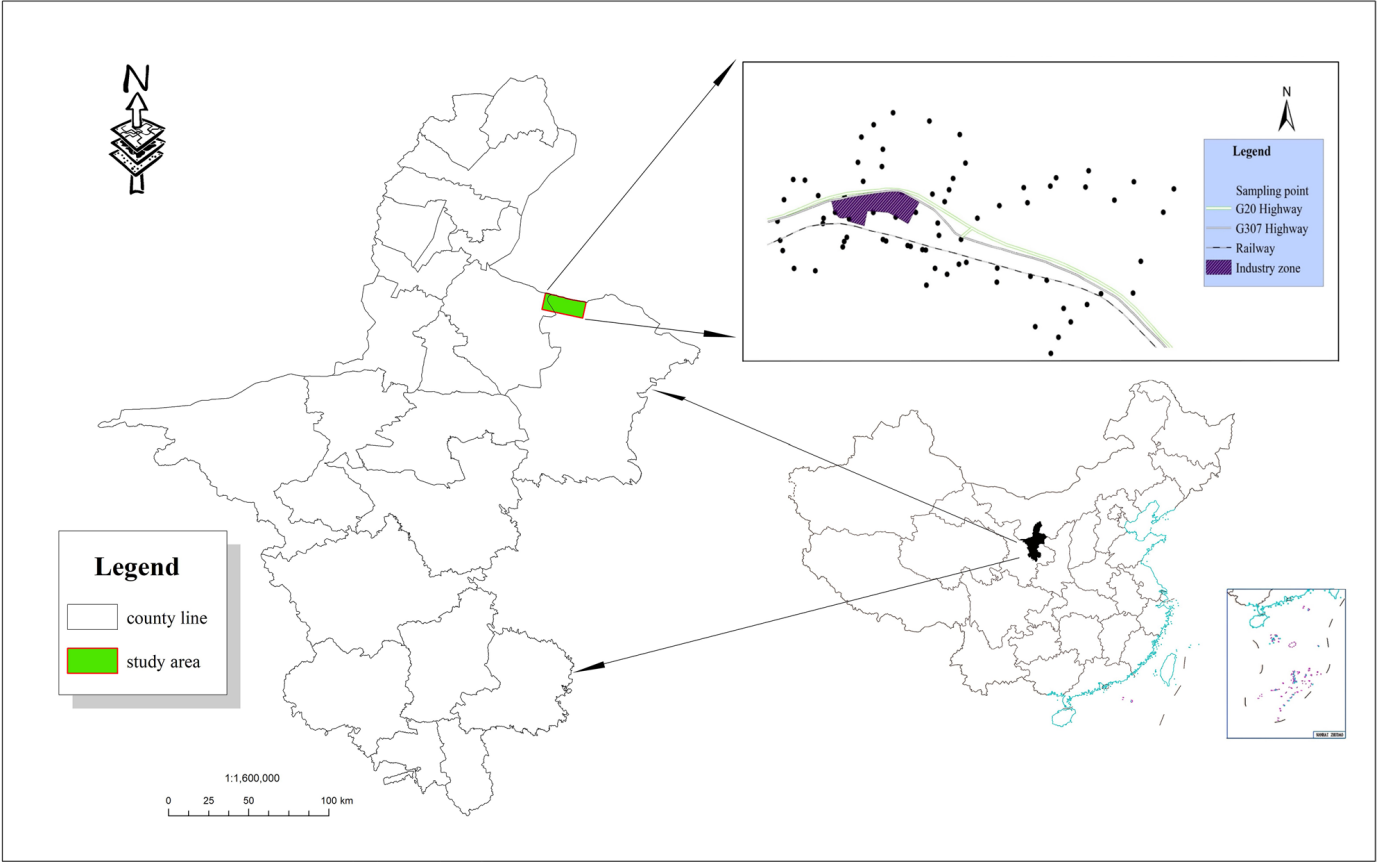
Distribution of soil sampling points in the study area near Gaoshawo town, Yanchi County, Ningxia, China (refer to Xu et al.^30^) (created by using ArcGIS Desktop 10.5, ESRI, California, US. https://desktop.arcgis.com).

In late June 2019, a field survey based on a guide for grassland resources and a comprehensive analysis of the slope, soil types, topographic features, landscape features, and accessibility of the industrial park were conducted. In different directions and at different distances from the industrial park, an interval of 1–2 km was used. A five-point cross sampling method was used to sample the surface soil (0–20 cm), and 5 soil samples from each plot were mixed. The original weight of the sample was greater than 1 kg, and a total of 82 soils were collected (Fig. 1). During field sampling, appropriate adjustments were made according to the actual environment around the preset sampling points^26^. According to the technical specifications for soil environmental monitoring (HJ/T166-2004), metal sampling equipment should not contact the soil during sampling, as this will affect the later detection of soil heavy metal content. The latitude and longitude of each sample point were recorded by GPS during sampling, and further environmental information around the sample point was recorded^27^. After the soil was air-dried, roots, rocks and other debris were removed, and the soil was ground through a 1 mm nylon sieve and bagged for later use. The determination of heavy metal elements requires a 100-mesh nylon sieve, and the soil was passed through 0.149 mm, 0.25 mm and 0.5 mm aperture sieves for the determination of physical and chemical properties. Three groups of parallel experiments were carried out, and the average value was taken. Samples were analysed according to methods described by Bao^28^ in Soil Agro-chemistry Analysis. The Kjeldahl method was used to determine the total nitrogen (TN) concentration. Near infrared spectroscopy was used to determine the total phosphorus (TP) concentration. Sodium bicarbonate extraction with molybdenum antimony resistance colorimetry was used to determine available phosphorus (AP). A flame photometric method was used for measuring available potassium (AK). The potassium dichromate method was used to determine soil organic matter (SOM), and the electric potential method was used to measure the pH value (the soil:water ratio was 5:1). Heavy metal concentrations in 0.5 g air-dried soils were digested using the HClO_4_–HNO_3_–HF method^29^, with a standard solution concentration gradient of 0.01, 0.1, 0.2, 0.5, 1, 5, and 10 μg·mL^−1^ and inductively coupled plasma-atomic emission spectrometry (HK-8100 ICP-AES Beijing Huake Yitong Analytical Instrument Co., Ltd.). The detection limits of copper (Cu), cadmium (Cd), chromium (Cr), lead (Pb), and zinc (Zn) were 0.003, 0.003, 0.005, 0.03, and 0.003 mg·kg^−1^, respectively. Experiments were repeated in triplicate, and the mean value was taken as the heavy metal concentration. The Chinese National Soil Standard Value (GSS-8) was used for quality control and recovery calculations to improve the accuracy of the analysis (Cu: 24.3 ± 1.2, Cd: 0.13 ± 0.02, Cr: 68 ± 6, Pb: 21 ± 2, Zn: 68 ± 4). The recoveries of the five heavy metals were 98–102% (Cu), 90–100% (Cd), 94–104% (Cr), 98–107% (Pb), and 96–105% (Zn). In addition, random checks and abnormal point checks were performed to ensure that the results met the quality control requirements^30^.

### Soil quality evaluation

Based on the IFI and PN^31^, all the indexes were combined into a more comprehensive index, the SQI, which was used to analyse the soil quality of the desert steppe in the study area.

#### Soil fertility quality evaluation

Based on the research of Hu et al.^32^, principal component analysis and membership functions were used to determine the weight and membership degree of the evaluation indexes. Due to the spatial variability in soil indicators and dynamic effects on the evaluation of soil fertility, the original data were standardized. The membership function model of each index was established to represent the state value of each evaluation factor^5^. According to observations in the study area, soil TN, TP, AP, AK and SOM belong to the membership function of Eq. (1):

1 $$\begin{array}{*{20}c} {f\left( {x_{i} } \right) = \frac{{\left( {x_{ij} - x_{imin} } \right)}}{{\left( {x_{imax} - x_{imin} } \right)}}} \\ \end{array}$$

where $f_{\left( x \right)}$ represents the subordination value of soil fertility evaluation index $i$ (0–1) and $x_{ij}$ represents the measured value of the soil fertility degradation evaluation index, and $x_{imax}$ and $x_{imin}$ represent the maximum and minimum observed values of soil fertility evaluation index $i$, respectively.

Using principal component analysis, principal components were calculated for the fertility parameters, with each axis representing a cumulative variance contribution rate, and then the weighted indexes of the soil fertility evaluation were calculated ($W_{i}$).

2 $$\begin{array}{*{20}c} {W_{i} = \frac{{Cap_{i} }}{{\mathop \sum \nolimits_{i = 1}^{n} Cap_{i} }}} \\ \end{array}$$

where $W_{i}$ represents the weight of soil fertility evaluation index $i$ in a given principal component $, Cap_{i}$ represents the absolute value of the contribution of soil fertility evaluation factor $i$ to a certain component, and $n$ represents the number of soil fertility evaluations.

Fertility status is determined according to the comprehensive evaluation value of each index. The IFI value is calculated using multiplication, and the formula is:

3 $$\begin{array}{*{20}c} {IFI = \mathop \sum \limits_{j = 1}^{m} K_{j} \left( {\mathop \sum \limits_{i = 1}^{n} W_{i} \times f_{{\left( {x_{i} } \right)}} } \right)} \\ \end{array}$$

where $n$ is the number of fertility index evaluations, $m$ is the number of selected principal components, $K_{j}$ is the variance contribution rate of principal component $j$, $f_{{\left( {x_{i} } \right)}}$ represents the degree of the membership function of soil fertility evaluation index $i$, and $W_{i}$ is the weight of soil fertility evaluation index $i$ in the principal component.

#### Evaluation of soil heavy metal pollution

Five heavy metal elements (Cu, Cd, Cr, Pb, and Zn) were selected for the pollution evaluation indexes, and a single-factor pollution index ($P_{i}$) and the PN were used for the evaluation^8^ according to the following equations:

4 $$\begin{array}{*{20}c} {P_{i} = C_{i} /C_{1} } \\ \end{array}$$

5 $$\begin{array}{*{20}c} {PN = \sqrt {\frac{{\left( {\overline{{P_{i} }} } \right)^{2} + \left( {P_{{i{\text{max}}}} } \right)^{2} }}{2}} } \\ \end{array}$$

In Eq. (4), $P_{i}$ is the pollution index of heavy metal element *i* in the soil, $C_{i}$ is the measured concentration of that heavy metal (in mg·kg^−1^), and $C_{1}$ is the soil pollution risk value of that element in agricultural land (mg·kg^−1^) according to the “Soil Environmental Quality Agricultural Land Soil Pollution Risk Control Standard” (GB 15618-2018II). In Eq. (5), $\overline{{P_{i} }}$ is the average value of the single-factor pollution index for all five metals, and $P_{{i{\text{max}}}}$ is the largest single-factor pollution index value.

#### Comprehensive evaluation of the soil quality index

A total of 10 evaluation indicators, including comprehensive soil fertility indicators and heavy metal elements, were used to analyse the soil quality of the desert steppe. The SQI comprehensive evaluation method of soil quality (Eq. 6) was used:

6 $$SQI = \left\{ {\begin{array}{*{20}l} 0 \hfill &\quad {PI_{ave} > 1} \hfill \\ {\sqrt {(SFI_{min}^{2} + SFI_{ave}^{2} )/(PI_{max}^{2} + PI_{ave}^{2} )} } \hfill &\quad {0.3 < PI_{ave} \le 1} \hfill \\ {1.5 \times \sqrt {(SFI_{min}^{2} + SFI_{ave}^{2} )} } \hfill &\quad {PI_{ave} \le 0.3} \hfill \\ \end{array} } \right.$$

where *SFI* is the integrated fertility index, $SFI = S_{i} /S_{1}$ is the calculation method, $S_{i}$ is the measured value of soil fertility, $S_{1}$ is the lower limit of the richness standard of each index of soil fertility according to the "DZ/T 0295–2016 Land Quality Geochemical Evaluation Code", $SFI_{min}$ is the minimum value of SFI, $SFI_{max}$ is the maximum value of SFI, $SFI_{ave}$ is the average value of *SFI*, $PI = C_{i} /C_{1}$, and $C_{i}$ and $C_{1}$ have the same values as those described in Eq. (4). The application of the *PI* discriminant value (0.3) is considered when various elements exceed the standard values and were obtained without pollution^33^.

With reference to Abd-Elwahed^34^ soil quality classification standards and observations in the study area, the SQI was divided into five grades, as shown in Table 1.

**Table 1:** Comprehensive soil quality index (SQI) classification grades.

SQI	Class
0.00–0.39	Very low
0.40–0.54	Low
0.55–0.69	Moderate
0.70–0.84	High
0.85–1.00	Very high

#### Spatial variability analysis

A Kolmogorov–Smirnov (K-S) test was performed on all the samples, and the data that were not normally distributed were converted with a Box-Cox transformation to ensure that they had an approximately normal distribution. Through the geostatistical analysis in the GS^+^9.0 software, the most suitable semi-variance function model was selected based on the criteria that the residual error (RSS) was close to 0 and the decisive factor (R^2^) was closest to 1; the larger the residual and the smaller the difference, the better or worse the model fit. Geostatistics were used to analyse the spatial variability in the soil fertility indicators and heavy metal elements with the following equation:

7 $$\begin{array}{*{20}c} {r\left( h \right) = \frac{1}{2N\left( h \right)}\mathop \sum \limits_{i = 1}^{N\left( h \right)} \left[ {Z\left( x \right) - Z\left( {x + h} \right)} \right]^{2} } \\ \end{array}$$

here $r\left( h \right)$ is the semi-variance function, $h$ is the lag distance,$N\left( h \right)$ is the logarithm of the data points separated by $h$, $Z\left( x \right)$ is the regionalized variable where the measured value is $x$, and $Z\left( {x + h} \right)$ is the measured value at $x + h$ for the regionalized variable.

### Data processing and statistical analyses

IBM SPSS Statistics 22.0 and Microsoft Office Excel 2019 software were used to conduct descriptive statistical analyses of the desert steppe soil quality indicators, GS^+^ 9.0 software was used for the semi-variance analysis of the soil quality indicators, and ArcGIS 10.5 was used to produce the kriging interpolation map.

### Ethics approval and consent to participate

This manuscript does not report on or involve the use of any animal or human data or tissue.

### Consent for publication

This manuscript does not contain data from any individual person.

## Results

### Analysis of the characteristics of the quality indicators

#### Descriptive statistics

Table 2 shows that the coefficient of variation of the soil pH in the study area was the smallest (0.03) and showed weak spatial variation. The average pH value was 8.13 ± 0.24, and the soil was alkaline. The average values of TN, TP, and SOM were 0.44 ± 0.19, 0.19 ± 0.06, and 11.08 ± 6.57 g·kg^−1^, respectively, and the average values of AP and AK were 8.4 ± 3.04 and 56.85 ± 25.31 mg·kg^−1^, respectively. The desert steppe has suffered severe desertification. The plant coverage is low, the plant growth is poor, there is a small amount of biomass, and the area has been subjected to human disturbance for a long time, which further caused generally concentrations of TN, TP, and AP in the study area. The coefficient of variation of the integrated fertility index in the study area ranged from 0.32–0.59; the exception was pH, which showed moderate variation. There was a spatial difference between the indexes, which may be related to dust diffusion from industrial transportation.

**Table 2:** Descriptive statistical characteristics of the soil quality indicators.

Quality index	Max	Min	Mean	Standard deviation	CV	Background values	GB 15618-2018II pH > 7.5/mg·kg^−1^	Classification standards from the second national soil survey^39^					
								I	II	III	IV	V	VI
TN (g·kg^−1^)	0.91	0.07	0.44	0.19	0.43	–	–	> 2	1.5–2	1–1.5	0.75–1	0.5–0.75	< 0.5
TP (g·kg^−1^)	0.49	0.11	0.19	0.06	0.32	–	–	> 1	0.8–1	0.6–0.8	0.4–0.6	0.2–0.4	< 0.2
AP (mg·kg^−1^)	17.40	5.40	8.40	3.04	0.36	–	–	> 40	20–40	10–20	5–10	3–5	< 3
AK (mg·kg^−1^)	155	23	56.85	25.31	0.45	–	–	> 200	150–200	100–150	50–100	30–50	< 30
SOM (g·kg^−1^)	45.51	1.38	11.08	6.57	0.59	–	–	> 40	30–40	20–30	10–20	6–10	< 6
pH	9.00	7.43	8.13	0.24	0.03	–	–	–	–	–	–	–	–
Cu (mg·kg^−1^)	30.30	13.00	21.64	3.26	0.15	22.1	100	–	–	–	–	–	–
Cd (mg·kg^−1^)	0.21	0.13	0.18	0.02	0.09	0.11	0.6	–	–	–	–	–	–
Cr (mg·kg^−1^)	101.90	18.20	44.99	21.23	0.47	60.6	250	–	–	–	–	–	–
Pb (mg·kg^−1^)	162.90	31.30	87.18	25.84	0.30	20.6	170	–	–	–	–	–	–
Zn (mg·kg^−1^)	169.80	42.60	85.63	24.98	0.29	58.8	300	–	–	–	–	–	–

*Max* maximum value, *Min* minimum value, *CV* coefficient of variation, *TN* total nitrogen, *TP* total phosphorus, *AP* available phosphorus, *AK* available potassium, *SOM* soil organic matter, *Cu* copper, *Cd* cadmium, *Cr* chromium, *Pb* lead, *Zn* zinc. The measured heavy metal data come from Xu et al.^30^.

GB 15,618-2018II: "Soil Environmental Quality in Agricultural Land Soil Pollution Risk Control Standards", soil pollution risk screening value from the State Environmental Protection Administration of China (2018).

Background value: CNEMC^35^ (China National Environmental Monitoring Center) (1990).

State Administration of Environmental Protection. Soil Environmental Quality Standard. Beijing: China Standards Press, 1995 (in Chinese).

In addition, the average values of the heavy metal elements Cu, Cd, Cr, Pb, and Zn were 21.64 ± 3.26, 0.18 ± 0.02, 44.99 ± 21.23, 87.18 ± 25.84, and 86.63 ± 24.9 mg·kg^−1^, respectively, which were all lower than the "Soil Environmental Quality in Agricultural Land Soil” standard values of soil pollution risk established in the Standard for Pollution Risk Control (GB 15618-2018II). The background values of Cu, Cd, Cr, Pb, and Zn in the study area exceeded the standard rates in Ningxia by 31.17%, 100%, 19.48%, 100%, and 84.42%, respectively. Metal accumulation had already occurred in the soil^30^. Cadmium and Pb may be strongly affected by human activities, as indicated by the fact that the contents of these elements exceeded the standard value by 100%. The order of the coefficients of variation of the heavy metals was as follows: Cr > Pb > Zn > Cu > Cd. Among these, the minimum coefficient of variation (CV) was observed for Cd (0.09), indicating weak variation. Taken together, these results suggest that the soil in the study area was contaminated with heavy metals, the soil fertility was low, and the values fluctuated greatly among the sampling points. This suggests that pollution may come from point sources during the discharge of wastes from industrial production.

#### Analysis of spatial characteristics

The semi-variance function model and the various quality indicators of desert steppe soil showed that Cr conformed to the Gaussian model; TP, SOM, and Cu conformed to the index model; and the other quality indicators conformed to the spherical model (Table 3). Except for AP (R^2^ = 0.497), the best theoretical models had a good model fit (R^2^ > 0.548). The residual range of all indicators in the study area was 1.01 $\times$ 10^–7^–0.517, and the fitting effect was satisfactory. The nugget value $(C_{O} )$ represents the variation caused by random factors. The study nugget value was between 0.0003 and 0.0642, indicating that the random variation due to measurement errors or soil properties was small. The abutment value $C + Co$ is the total spatial variability in the regional variables in the study area and is affected by environmental factors. The abutment value of the soil quality index in the study area ranged from 0.003 to 0.2828. The nugget coefficient ($C_{O} /Co + C$) can distinguish the spatial correlation of soil indicators^36,37^. For the nugget coefficients of TN, TP, AP, SOM, Cu, and Cd, the coefficients were between 0.339% and 23.637%; values less than 25% were mainly affected by structural factors (climate, parent material, topography, etc.) and show strong spatial autocorrelation^38^. The nugget coefficients of AK, Cr, Pb, and Zn ranged from 30.79% to 47.35%, and the spatial correlation of these elements was moderately strong; the nugget coefficients of these elements was affected by both structural and random factors. The research area is located in an important industrial transportation area with intensive industrial production activities; as a consequence, the high input of heavy metals and other pollutants into the surrounding environment has disrupted the health and stability of the soil in the research area.

**Table 3:** Theoretical model and related parameters of the semi-variance function of the soil quality index.

Quality index	Model	Nugget value $(C_{O} )$	Abutment value $C + Co$	Nugget coefficient $C_{O} /Co + C$%	Decisive factor R^2^	RSS
TN (g·kg^−1^)	S	0.009	0.005	17.796	0.717	2.672 $\times$ 10^−6^
TP (g·kg^−1^)	E	0.002	0.052	3.650	0.602	3.232 $\times$ 10^−4^
AP (mg·kg^−1^)	S	0.0003	0.089	0.339	0.548	1.997 $\times$ 10^−3^
AK (mg·kg^−1^)	S	0.058	0.160	36.267	0.703	2.882 $\times$ 10^−3^
SOM (g·kg^−1^)	E	0.029	0.283	10.396	0.479	3.623 $\times$ 10^−2^
Cu (mg·kg^−1^)	E	0.004	0.017	23.637	0.532	1.673 $\times$ 10^−4^
Cd (mg·kg^−1^)	S	0.002	0.008	22.368	0.800	4.809 $\times$ 10^−6^
Cr (mg·kg^−1^)	G	0.057	0.185	30.790	0.695	6.129 $\times$ 10^−3^
Pb (mg·kg^−1^)	S	0.021	0.043	47.349	0.578	3.986 $\times$ 10^−4^
Zn (mg·kg^−1^)	S	0.064	0.183	35.140	0.563	0.517

*RSS* residual error, *TN* total nitrogen, *TP* total phosphorus, *AP* available phosphorus *AK* available potassium, *SOM* soil organic matter, *Cu* copper, *Cd* cadmium, *Cr* chromium, *Pb* lead, *Zn* zinc, *S* spherical model, *E* exponential model, *G* Gaussian model.

### Analysis of soil quality characteristics of the desert steppe study area

#### Evaluation of soil fertility quality

To further explore the soil fertility status of the desert steppe, before principal component analysis, the soil fertility index was processed without dimensions to obtain the normalized membership degree value. SPSS software was used to conduct principal component analysis on soil fertility indicators, and the cumulative variance contribution rate of the three principal components was 75.843% (Table 4), which reflects most of the variance in the study area. It is generally believed that the greater the factor load is, the greater the weight of the variable in the corresponding principal component. Based on the loading of each factor in the principal components, the weights of different principal components are calculated^39^. The non-parametric Kolmogorov–Smirnov test (Table 5) showed that the soil fertility index data were normally distributed (*P* > 0.05). The 82 soil samples from the desert steppe study area had IFI values of 0.083–0.409, with an average value of 0.211 and a coefficient of variation of 0.326, which indicated moderate variation. The soil fertility in the study area was low.

**Table 4:** Weights of principal component contributions and soil fertility quality indexes.

Evaluation index	PCA1		PCA2		PCA3	
	Capacity	Weight	Capacity	Weight	Capacity	Weight
TN	0.722	0.308	0.436	0.224	0.023	0.018
TP	0.617	0.263	0.382	0.196	0.042	0.033
AP	0.098	0.042	0.906	0.465	0.024	0.019
AK	0.809	0.345	0.200	0.102	0.221	0.170
SOM	0.097	0.042	0.025	0.013	0.985	0.760
Variance contribution rate %	39.067		21.538		15.238	
Cumulative variance contribution rate %	39.067		60.605		75.843	

*TN* total nitrogen, *TP* total phosphorus, *AP* available phosphorus, *AK* available potassium, *SOM* soil organic matter.

**Table 5:** Comprehensive descriptive statistics of soil quality in the study area.

Project	Max	Min	Mean	SD	CV	Skewness	Kurtosis	K-S test
$IFI$	0409	0.083	0.211	0.069	0.326	0.929	0.075	0.200
$\it {\text{PN}}$	0.734	0.237	0.422	0.104	0.246	0.344	− 0.198	0.200
$SQI$	0.633	0.252	0.403	0.088	0.217	0.600	0.286	0.200*

*After logarithmic conversion. *Max* maximum, *Min* minimum, *SD* standard deviation, *CV* coefficient of variation, *K-S* Kolmogorov–Smirnov, *IFI* integrated fertility index, *PN* Nemerow pollution index, *SQI* soil quality index.

Through the IFI spatial distribution map (Fig. 3-IFI), the overall status of the soil fertility can be more intuitively understood. Most integrated fertility indexes in the study area were between 0.18 and 0.25. The areas with a high fertility index were distributed in the southeastern part of the industrial park (on both sides of the highway) and showed a patchy spatial distribution.

#### Evaluation of soil heavy metal pollution

**Figure 2:**
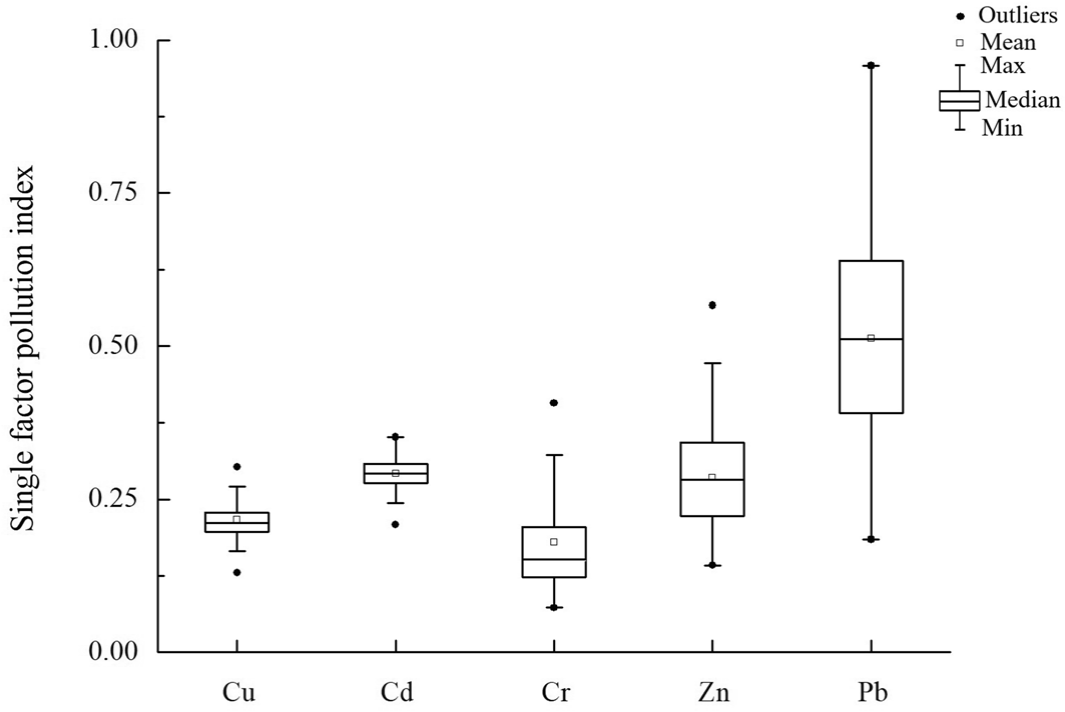
Single-factor pollution indexes for the heavy metals copper (Cu), cadmium (Cd), chromium (Cr), zinc (Zn), and lead (Pb) averaged from soil samples.

Cu, Cd, Cr, Zn, and Pb were selected as heavy metal pollution indicators, and China's second level standard of soil environmental quality (GB 15618-2018II) was used as a reference to evaluate the status of heavy metal pollution in the study area. The PN was between 0.237–0.734, and the average pollution index was 0.422. The larger the PN is, the more severe the pollution of heavy metals in the soil. When a single pollution index was used (Fig. 2), Pb was identified as the main heavy metal causing pollution in the study area.

**Figure 3:**
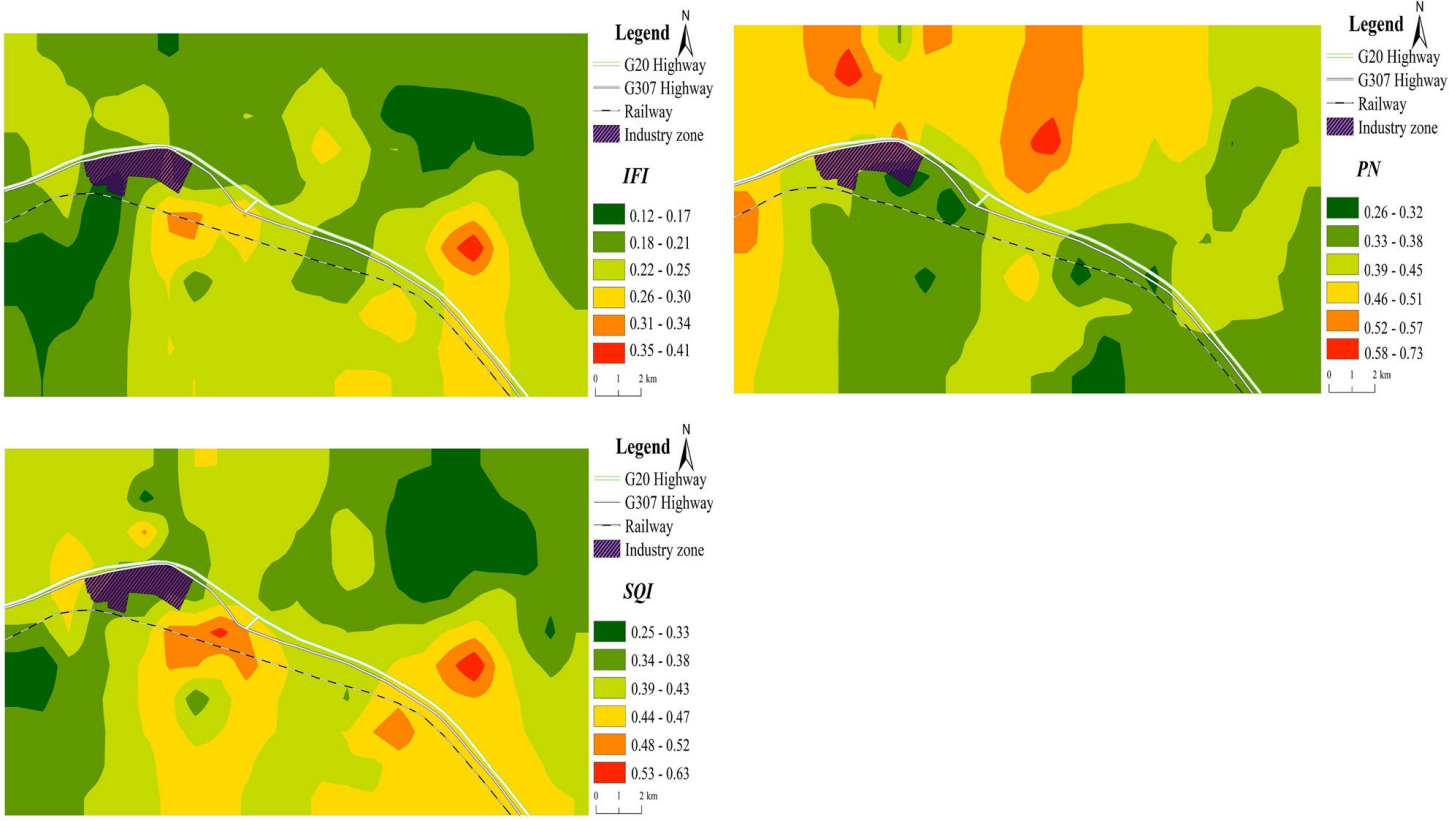
Spatial distribution of the integrated fertility index (IFI), Nemerow pollution index (PN) and soil quality index (SQI) in the study area (created by using ArcGIS Desktop 10.5. ESRI, California, US. https://desktop.arcgis.com).

Combined with the spatial distribution map (Fig. 3-PN), these results indicated that the area with a higher pollution index was patchily distributed in the area north of the highway. Pb and Cd were the key contributors to this phenomenon, which was probably because the study area is the main area of industrial transportation in the region. Coal slag transported by coal trucks and car exhaust generates dust pollution, which contributes to pollution around highways. Although the use of leaded gasoline has been banned in China since 2000, a large amount of Pb remains in the soil^10^, and the Pb concentration in the soil of the study significantly exceeds background concentrations. Cadmium is often used as an indicator of agricultural activities, such as the application of pesticides and fertilizers^37^. The Ming Great Wall, present in the northern part of the study area, is not only an ancient military fortress but also an important geographical indicator of China’s agricultural and pastoral zone. In recent years, the livestock breeding industry along the Great Wall has rapidly developed, and the accumulation of livestock manure and domestic waste has increased the Cd content in the area.

#### Comprehensive evaluation of soil quality

The integrated fertility index and contents of the heavy metal elements were combined to calculate the SQI of the desert steppe in the study area. The results showed that the SQI data conformed to a normal distribution (Table 5), the coefficient of variation was 0.217, indicating moderate variation, and the range of variation in the SQI was 0.252–0.633, with an average value of 0.403. According to the SQI classification grades in Table 1, the soil quality in the study area was extremely low.

The SQI distribution map of the study area (Fig. 3-SQI) shows that most of the area was dominated by SQI values of 0.34–0.47, while some areas showed lower index values. The areas with higher quality index values were patchily distributed in a southeasterly direction, which is similar to the spatial distribution of the soil IFI and opposite to the spatial distribution of the PN. In general, the IFI, PN, and SQI had regularities in their spatial distributions, which were related to the topography and dominant wind direction of the study area. There are many coal plants in the industrial park. Disturbances to the surrounding soil and the release and deposition of coal ash, dust, heavy metal elements, etc. from these plants are affected by prevailing winds. The northwesterly wind causes particles to settle in the low-lying areas north of the highway, reducing the soil fertility and quality in that area. Therefore, the soil quality in the southeastern part of the study area is high, and the deposition of heavy metals and other pollutants across the study area is the key factor causing this phenomenon.

**Figure 4:**
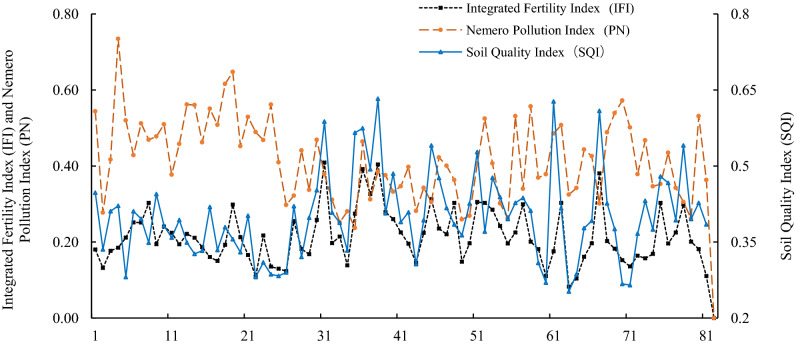
Comparative analysis between the three soil quality evaluation methods.

In the correlation analysis (Table 6), the IFI was significantly positively correlated with the SQI (*P* < 0.01), and the PN was negatively correlated with the IFI and SQI. This analysis, combined with the results shown in Fig. 4, indicate that the SQI and PN followed an intuitive pattern on the comprehensive soil quality index curve. As the IFI increased, the overall trend of the SQI increased, although there were fluctuations. At the inflection point of the fluctuation, the relationship between the PN and SQI was evident. The PN, as a negative indicator of soil quality, is the inverse of the IFI and SQI. When pollution was present, the PN increased and the SQI decreased; when the PN decreased, the SQI increased and the soil was considered unpolluted. The IFI is a positive indicator of soil quality, and changes in this index were consistent with those in the SQI. The values of the SQI were mainly between those of the IFI and PN, which showed both the positive and negative effects of soil fertility and heavy metal pollution on soil quality and demonstrated that the comprehensive evaluation of soil quality can better reflect the impact of soil fertility and heavy metal pollution on soil quality^22,40^.

**Table 6:** Correlation analysis between IFI and PN.

Element	IFI	PN	SQI
IFI	1		
PN	− 0.069	1	
SQI	**0**.713**	− 0.165	1

*Indicates *P* < 0.05, and the correlation is significant. **Indicates *P* < 0.01, the correlation is extremely significant.

## Discussion

### Evaluation of soil fertility in a desert steppe

Soil is an important component of grassland ecosystems and is an essential carrier of material and energy. Soil nitrogen, phosphorus, and potassium are the main mineral elements required for plant growth and development. These nutrients play a vital role in plant nutrient cycling, physiology, and biochemistry and are key indicators of soil quality^40–43^. In this study, TN, TP, AP, AK, SOM, and pH were selected to reflect soil fertility. Compared with the background soil fertility status of China, the IFI of the study area was relatively poor, at 0.393. With the exception of AP, the nugget coefficients of the fertility indicators were less than 25%, which suggests that they were affected by natural factors and showed a strong autocorrelation. There are large differences in soil between northern and southern China, with soil in the south being more acidic and having higher SOM^44^. The current study focused on the northern desert steppe zone, where there is perennial drought and little rain, severe desertification, and water and nutrient shortages and the soil water holding capacity is low, all of which contribute to low concentrations of soil nitrogen, phosphorus, other nutrients, and SOM. Ding et al.^45^ showed in their study of the herb layer of the Gurbantunggut Desert in northwestern China that similar natural environmental factors were the main reason for low soil nutrients.

In the current study, the coefficient of variation of the soil SOM was 0.59, which may be related to the coverage of soil by coal dust. The highways in the study area are mainly used to transport coal-based raw materials. The annual traffic flow is high, which accelerates dust generation. Moreover, this leads to a larger nugget coefficient of AK, because AK has a relatively weak adsorption capacity and high mobility^46^ and eventually enters soil. As a result, AK did not follow the same spatial patterns as the other soil fertility indicators.

Studies have pointed out that long-term accumulation of dust on the soil surface leads to changes in soil nutrients^47,48^. In India, coal mining and coal transportation are responsible for degrading the soil quality of nearby farmlands^49^. However, in the mining area near the Xilinguole grassland in northern China, dust and ash had a regulating effect on soil nutrients and increased the concentrations of soil AP and AK^50^. From the radar chart of the average membership degree, we observed that SOM was the main factor limiting soil fertility in the current study. As the largest contributor to soil fertility, it plays an important role in maintaining productivity^51^. The reduction in the SOM input and the increase in soil disturbance resulted in a decrease in other nutrients in the study area^52^. Liu et al.^53^ found at the Huitengxile Wind Farm in northern China that dead roots and vegetative litter were transformed into SOM in soil by microbial decomposition, which caused the mineralization process to decline and, in turn, decreased soil AP and AK. Studies have also shown that livestock excreta^54^ and trampling^55^ are important for regulating soil nutrients. Most of the ingested plant tissues return to the grassland through the excreta, accelerating soil-nutrient cycling^56^. Farms were distributed on both sides of the highway in the study area, and their impact on soil fertility cannot be ruled out.

The IFI spatial distribution map showed that the soil quality in the southeastern part of the study area was relatively good. This was largely because the pollutants generated from transportation and industrial production in the area were affected by the terrain and prevailing wind direction, accumulating in the low-lying areas north of the highway. Many of these pollutants were heavy metals, which reduced the integrated fertility index. Therefore, the soil fertility in the southeastern region was better due to the spatial distribution of pollutants, although natural factors were still the main cause of the low soil fertility. It is undeniable that industrial activities have caused damage to grassland ecosystems and soil health^57^.

### Current status of heavy metal pollution in desert steppe soil

Soil pollution caused by heavy metals is one of the most urgent environmental problems in the world. In the current study, soil Cu and Cd exceeded background concentrations in the study area by 31.17% and 100%, respectively, and the nugget coefficients were less than 25%, indicating that this pattern was mainly due to structural factors (climate, parent material, terrain, etc.). Impact studies have shown that dust and waste generated from coal mining activities contain large amounts of sulfides and disulfides, which are the main sources of Cu and Cd pollution^58^, increasing soil concentrations of Cu and Cd as a result. However, agricultural production is also a significant source of Cu and Cd^59^ because fertilizers and pesticides can contain large amounts of these metals. In fact, long-term fertilization was identified as the main cause of soil Cd accumulation^60^. Therefore, industrial and agricultural activities, in addition to natural factors, are the probable causes of Cu and Cd pollution in the study area. There was a moderate spatial correlation among the Cr, Pb and Zn nugget coefficients, which were between 30.79% and 47.35%. Chromium exceeded the background concentration by 19.48%, and the coefficient of variation was large, indicating that its concentrations were influenced by both natural and human factors. At present, most industrial activities rely on coal combustion, which oxidizes Cr6^+^ compounds at high temperatures during the production process. Increasing amounts of insoluble compounds, including Cr6^+^, are deposited in soil and undergo limited vertical migration, resulting in heavy metal accumulation in surface soils^61^. In the semi-arid area of Iran, industrial and agricultural activities have led to the excessive accumulation of heavy metals in the local soil^62^.

The PN evaluation method combines single-factor pollution indexes and the comprehensive pollution index method to thoroughly reflect the pollution status of a study area. Huang et al.^63^ highlighted the impact of pollutants with the largest pollution index on environmental quality and showed that heavy metal pollution in desert steppe soil was mainly affected by Pb. In industrial areas in northeastern and southwestern China, industrial activities are the main source of Pb pollution^64^. The general sources of Pb contamination in the environment are automobile exhaust and industrial emissions^65,66^; there are reports that car exhaust accounts for approximately two-thirds of global Pb emissions, causing regional Pb pollution through atmospheric deposition. In addition, coal mining and combustion emissions may also be important contributors to Pb via atmospheric deposition^67^. Copper and Zn are sulfurphilic elements and are also present in motor vehicle emissions. They have strong homology and change in a consistent manner under similar external conditions^68^. Adachi and Tainosho^69^ suggested that as ZnO was added as a catalyst in the hardening stage during the tire-making process, Zn can be released into the environment as tires become worn. A large amount of Zn was detected in soil near a steel plant in Madrid, Spain^70^. In the current study, the Zn content exceeded the background standard by 84.42%, and its single-factor pollution index was second only to Pb. Thus, we suggest that Zn and Pb are derived from similar sources and are affected by the industrial activities and transportation in the region.

The PN spatial distribution map shows that pollution increased from south to north in the study area, largely due to the prevailing wind direction and topography. Fan et al.^71^ reached a similar conclusion in their study of the Ningxia Shizuishan Industrial Park. The focus of industries occupying the Gaoshawo Industrial Park is mainly on coal, mining materials, reagent manufacturing, and petrochemical processing. Pollutants generated by fuel combustion, soil dust, industrial smelting and other production processes were affected by the dominant wind direction, with deposition and accumulation occurring in the northern low-lying parts of the study area.

### Comprehensive evaluation of desert steppe soil quality

The interaction and coordination among the soil quality indicators reflects the level of soil productivity and adaptability to adversity and indicates the factors influencing soil quality. A soil quality evaluation is based on soil function and reflects the impact of natural factors and human activities on soil^72^. Industrial transportation, waste discharge and agricultural activities cause environmental pollution^7,73,74^. Masto et al.^49^ found that mining activities adversely affected soil in the agricultural areas surrounding a mining area, leading to changes in the physical and chemical properties of the soil. However, some scholars have shown that mining and other industrial activities can improve soil nutrients^75^ and that industrial activities can release large amounts of pollutants and trace elements^47,48,76^, resulting in changes to the SOM. SOM forms a complex with heavy metals through adsorption, which reduces the bioavailability of the metals. In contrast, low-molecular-weight organic matter in soil can act as a chelate and improve the bioavailability of metals^77^. Thus, human inputs can affect the physical and chemical properties of soil and control the concentration and bioavailability of heavy metals, which directly or indirectly affects the stability of the soil^78^.

The spatial distribution map of the SQI allowed the overall status of soil quality in the study area to be more intuitively understood. It more clearly showed the influence of topography and the prevailing northwesterly winds on the south to north distribution of pollutants. Here, the highway was taken as a demarcation point, with soil pollution increasing to the north and decreasing to the south. These pollutants would have been dispersed in the air as dust and accumulated over the landscape over time. The research area is near Yanchi County and the Ningdong Energy and Chemical Base. The development of the industries in the industrial park has resulted in an increase in the number of enterprises and traffic flow and, ultimately, has increased the vulnerability of the desert steppe to pollution. Given that little data exist on soil quality and the impacts of soil pollution on soil in the desert steppe, data from the current study will provide a reference for evaluating and protecting desert steppe ecosystems from soil pollution.

## Conclusion

This study used the correlation coefficient method, membership function and geostatistical methods to evaluate the soil fertility status and heavy metal pollution of a desert steppe region under industrial activities and provided a comprehensive assessment of the quality status of the soils in the region. The main conclusions are outlined below:

1. The average value of the IFI was 0.387, the soil was alkaline, and the fertility index was low. The PN was 0.421, and Pb was the key factor contributing to this value. The average SQI of the study area was 0.351, indicating that the desert steppe soil quality was extremely low. The PN was negatively associated with the IFI and SQI.
2. The spatial assessment of these indexes showed that pollutant distribution was affected by the terrain and wind direction, with heavy metals and other pollutants deposited north of the highway in the study area, thereby reducing soil quality in the north. It was evident that the industrial activities and transportation in the study area significantly impacted the desert steppe soil quality.

## Data Availability

The datasets generated during and/or analyzed during the current study are available from the corresponding author on reasonable request.
